# Sensory Analysis for Cow Milk Product Development Using High Pressure Processing (HPP) in the Dairy Industry

**DOI:** 10.3390/foods11091233

**Published:** 2022-04-25

**Authors:** Shu Huey Lim, Nyuk Ling Chin, Alifdalino Sulaiman, Cheow Hwang Tay, Tak Hiong Wong

**Affiliations:** 1 Department of Process and Food Engineering, Faculty of Engineering, Universiti Putra Malaysia, UPM, Serdang 43400, Selangor, Malaysia; gs59164@student.upm.edu.my (S.H.L.); alifdalino@upm.edu.my (A.S.); 2 F&N Global Marketing Pte. Ltd., 438 Alexandra Road, 20-00 Alexandra Point, Singapore 119958, Singapore; justin.tay@fn.com.my (C.H.T.); takhiong.wong@fnnfoods.com (T.H.W.)

**Keywords:** raw milk, triangle test, acceptance and preference test, penalty values

## Abstract

High pressure processing (HPP) can be applied as an alternative thermal treatment of milk to maintain its natural and original sensory quality. Milk was processed at 600 MPa for 10 min or given thermal treatment at 125 °C for 4 s. Sensory evaluation of treated milk samples was conducted using the triangle and the acceptance and preference tests. The triangle test was used as a discriminative test to check whether there was a noticeable difference between both treated milk samples. The acceptance and preference test determined attributes of milk including colour, milkiness, creaminess, mouthfeel, and aftertaste based on the 5-point just-about-right (JAR) scale. In the triangle test, 89.5% of panellists were able to identify the odd sample and differentiate milk processed using high pressure from heat treatment. For the acceptance and preference test, 61% of panellists gave higher overall preference for the high pressure processed milk over heat-treated milk. The JAR evaluation showed no significant differences (*p* > 0.05) in all evaluated milk attributes which included milkiness, creaminess, mouthfeel, and aftertaste, with the exception of colour. Overall, high pressure processed milk scored better in terms of organoleptic properties as the penalty value for most attributes including colour, milkiness, mouthfeel, and aftertaste were lower than the penalty of heat-treated milk, except for creaminess. Therefore, to improve the acceptance and preference of high pressure processed milk, future development needs to focus on increasing creaminess of high pressure processed milk.

## 1. Introduction

Milk is a balanced and nutritive drink which is widely consumed by all age groups for health benefits alongside its pleasant aroma, mouthfeel, and slightly sweet taste [1]. The nature of milk and its chemical composition makes it one of the ideal culture media for microbial growth and multiplication [2]. To ensure safe consumption and to lengthen the shelf life of milk, different heat treatments are commonly applied to raw milk to remove pathogenic organisms and spoilage bacteria [3]. Thermal treatments such as pasteurisation using low temperature long time (LTLT, 63 °C for 30 min), high temperature short time (HTST, 72–75 °C for 15–20 s), ultra-pasteurisation (ESL, 125–128 °C for 2–4 s), and ultra-high temperature (UHT, 135–140 °C for 1–3 s) are widely used in the dairy industry [4,5]. Of these, only the UHT process is able to give milk products several months of shelf life at a room temperature storage condition [6]. The key factor that enables shelf stability for UHT products is the integration of UHT technology with aseptic processing where sterile milk leaving the heat exchanger is directly filled into sterile hermetically sealed packaging or containers to prevent recontamination [7,8]. Later development of thermal processing gave rise to ultra-pasteurisation technology, or extended shelf life (ESL) products, due to requirements to further increase the shelf life of pasteurised products to provide convenience and additional protection against temperature abuse [9]. Ultra-pasteurisation is the basis for extended shelf life which is capable of producing ESL milk with a long refrigerated storage life [10]. This process becomes a priority for some manufacturers who aim for a further 30–40 days of storage on top of the 2–16 days that are traditionally associated with pasteurised products.

Although thermal treatment is desirable from a microbiological point of view, it also causes adverse effects on nutritional quality, appearance, and flavour of milk [7]. Previous studies have established that heat treatments from 72–145 °C cause changes in sensory quality of milk where a cooked taste and smell or caramel flavour arise [11,12]. Lee et al. [13] reported changes in the sensory quality of milk with the development of tastes such as a cooked or caramelised flavour, sweet, bitter, and astringency as well as colour differences when milk is treated by the ultra-pasteurisation method at 138 °C for 2 s. Strong flavour intensities of heat-treated milk are undesirable and affect consumers’ acceptance [13,14].

The deficiency of heat treatment can be overcome using the non-thermal treatment technology of high pressure processing (HPP) [15]. HPP is a viable alternative to traditional thermal treatments because it can inactivate foodborne pathogens while avoiding nutrient loss, such as water-soluble vitamins, and preserving freshness of food products [16,17]. As part of raising awareness of healthy consumption, many food industries are working to improve existing products and develop new ones to satisfy consumers’ demands and preferences for natural, safe, and high-quality products. The HPP approach is a favoured processing method explored by food manufacturers because of its capability to produce natural food with an extended shelf life [15]. The HPP process is usually performed in the pressure range from 300–1000 MPa for a few minutes at room temperature [18,19,20]. The temperature may get a little higher as compression increases the temperature of food by approximately 3 °C per 100 MPa [21]. Studies have proven the effectiveness of HPP in reducing microbial content by at least 5 log CFU/mL [22,23] and in lengthening the shelf life of milk by at least 7 days when compared with heat-pasteurised milk [20]. There are many related works undertaken by researchers on high pressure treatment which have proven its potential in preserving dairy products while maintaining key quality characteristics similar to those of raw fresh milk [17,20,24,25].

With known positive effects of HPP treatment on milk, this study has focused on measuring the sensory attributes of HPP-treated milk in comparison with heat-treated milk in the effort of new product development. Sensory analysis is often used in food sectors to evaluate food products’ quality and consumer acceptance via an attribute assessment of texture, flavour, taste, appearance, smell, etc. using the senses of sight, smell, taste, and touch [16,26]. The trend of using sensory analysis in new product development in the food industry is documented by the European Institute of Innovation and Technology (EIT) Regional Innovation Scheme [27]. Sensory analysis is also critical for every application of milk and it is widely used by dairy manufacturers to identify deviation in processing and handling [28]. For comparative study of two nearly similar products, the triangle test is most commonly used in the dairy industry to verify whether two formulations are perceived to be different [28]. The result of this simple test can be analysed using binomial calculation where its *p*-value helps to determine the existence of a difference. The predefined statistical significance level for most difference tests is often set at *p* = 0.05. If the resulting *p*-value is less than 0.05, the null hypothesis is rejected and the products are believed to be distinct. Following a conventional approach of the triangle test, the number of observations *n* is fixed and each observation is independent [29]. When the number of observations is un-predefined and depends on the observations made progressively, it is known as the sequential analysis in ISO 16820:2019 [30]. This analysis is usually conducted for panel trainee selection [31,32] where the total number of correct response is plotted against the number of total tests performed [32]. The triangle test has been used by various researchers in milk studies. Lynch et al. [33] used the triangle test to check whether pasteurised milk samples with an elevated amount of conjugated linoleic acid and vaccenic acid were noticeably different in taste. Hanson and Metzger [34] conducted the triangle test to determine whether consumers could detect differences in samples fortified with increased amount of Vitamin D. Bandla et al. [35] used the triangle testing method to verify whether UV-C processing of raw cow milk treated in a continuous flow coiled tube ultraviolet reactor resulted in perceived changes to the taste of milk. Bottiroli et al. [36] conducted the triangle test to determine whether perceptible sensory differences exist among ultra-high temperature hydrolyse-lactose milk stored at different temperatures.

A sensory test frequently consists of an acceptance and preference test to obtain consumer perspective on the end product. This evaluation allows manufacturers to understand consumers’ likes, dislikes, and preferences before spending a large amount of capital on the production of a new product or investing in a new technology. The acceptance test is a direct evaluation of an individual product, typically using a type of scale to quantify overall acceptability [37]. Information collected from an acceptance test can be analysed through parametric statistical analysis and data generation [38]. The preference testing requires selection of the preferred product through ranking methods or direct questions [37]. This method permits identification of sample preference within the test set and from there allows evaluation of the marketability of the new product [38]. The just-about-right (JAR) scale combined with a hedonic scale is commonly used in the acceptance and preference test to measure the appropriateness level of an attribute and to test consumer preference and acceptability of foods [39,40,41]. JAR scales are bipolar labelled attribute scales with a midpoint anchored with “just-about-right (JAR)” or two semantically opposite anchors, e.g., “too strong” and “too weak” [42,43,44]. Tribst et al. [45] used JAR and hedonic scales to indicate whether stirring and homogenisation have effects on the acceptance of milk products in terms of texture, flavour, acidity, and overall liking. Similarly, combination of JAR and hedonic scales were used by Dong et al. [46] in their investigation on the effects of augmented reality (AR) environments on sensory responses of consumers towards different dairy products. For attributes which did not meet the JAR level, the penalty analysis (PA), or mean drop analysis, is conducted to identify which attribute of the product deviated significantly from optimal levels. The PA allows the product developer to identify attributes that are most penalising to product performance, thus allowing improvement to be made on that attribute.

In general, the sensory quality of HPP milk has been reported to be better than that of heat-treated milk. The organoleptic properties of milk have improved with higher acceptability scores due to reduced proteolytic activity after undergoing high pressure treatments at 400 MPa for 15 min [47], higher aroma and acceptability of HPP milk treated at 450–650 MPa for 3 min over pasteurised milk [48], and a different aroma profile in HPP milk at 600 MPa for 5 min compared to LTLT milk (63 °C for 30 min) due to higher levels of ketones [49]. Andrés et al. [48] added that HPP-treated milk was almost similar to untreated milk. The objective of this study was to evaluate the sensory properties of HPP treatment of milk at 600 MPa for 10 min in comparison with thermal treatment at 125 °C for 4 s for overall milk acceptance and in terms of colour, milkiness, creaminess, mouthfeel, and aftertaste attributes.

## 2. Materials and Methods

### 2.1. Processing of Milk

Pressurised milk was compared to thermally processed milk using fresh and raw cow milk collected from the university farm, Ladang 16, Universiti Putra Malaysia. After collection, the sample milk was mixed evenly and cooled to 4 °C. Milk subjected to high pressure processing was transferred into 350 mL food grade polyethylene terephthalate (PET) bottles. The milk samples were pressurised in a high pressure processing unit (Hiperbaric 55; Hiperbaric High Pressure Technologies, Burgos, Spain) at 600 MPa for 10 min. The pressurisation process was completed at room temperature at 25 ± 2 °C. Milk subjected to thermal treatment was ultra-pasteurised at 125 °C for 4 s referring to the indirect heating method stated by Deeth [10]. The customised pasteuriser (Powerpoint International Ltd., Saitama, Japan) consists of a tubular heat exchanger system with 2 heating, 3 cooling, and 1 holding tube/s at a capacity of 20 LPH. Ultra-pasteurised milk was also transferred into 350 mL PET bottles at 6–6.5 °C. All milk samples were stored at 8 °C in a designated fridge before conducting the arranged sensory tests the next day after both treatments that were conducted on a same day. High pressure treated milk was labelled as HPP milk while ultra-pasteurised milk was labelled as ESL milk. Table 1 shows the main properties of untreated (raw) and treated milk (HPP and ESL).

### 2.2. Sensory Analysis

Sensory profiling was conducted by internal panellists from a dairy manufacturing company and Universiti Putra Malaysia staff with reference to the guidelines in ISO 8586:2014 stating the selection, training, and monitoring of assessors and expert sensory assessors [50]. The panellists selected were non-smokers, had the ability to perform sensory tasks, and had interacted in discussions of sensory attributes during a 10-min briefing session conducted to train the assessors to be familiar with the products, lexicon, and the prescribed sensory evaluation procedures. The 38 panellists who participated in this evaluation were from different ethnic groups (68% Chinese, 5% Malay, 24% Indian, and 3% Siamese). The sensory evaluation was divided into 2 different sessions, i.e., the triangle test and the acceptance and preference test. The samples of approximately 30 mL were served at a serving temperature of 17 °C. Plain water was provided for panellists to clean their palate between sample tasting. Standard fluorescent light was used during the evaluation.

#### 2.2.1. Triangle Test

The triangle test was conducted following the procedures in ISO 4120:2004 [51]. Three samples with different 3-digit random codes were presented simultaneously to the panellists. The serving method was based on randomisation with an assured two samples from the same processing method and one from the other method (e.g., set of 2 HPP and 1 ESL milk samples, or 2 ESL and 1 HPP milk samples). Each panellist had to taste all 3 milk samples and indicate which was the odd sample. The panellists were advised to rinse their mouth with plain water before evaluating each sample. After identifying the odd sample, each panellist was required to indicate the degree of difference between the odd and duplicate samples to the level of difference felt as “None”, “Slight”, “Moderate”, “Much”, or “Extreme” following the method in ISO 6685:2017 [52]. These qualitative data were converted to quantitative data as follows: None-0, Slight-1, Moderate-2, Much-3, and Extreme-4, and were analysed using descriptive statistics analysis to summarise the responses. The binomial table was used to determine minimum numbers of correct judgements to establish significant difference at desired probability levels for the triangle test.

#### 2.2.2. Acceptance and Preference Test

In the acceptance and preference test, the panellists were served with a new set of two samples consisting of 1 HPP and 1 ESL labelled with 3-digit random codes. Each panellist tasted and evaluated their overall liking using the 7-point hedonic scale followed by the acceptance and preference using the 5-point just-about-right (JAR) scale for 5 attributes: colour, milkiness, creaminess, mouthfeel, and aftertaste. For the 7-point hedonic scale evaluating overall liking, panellists rated each sample as 1 = dislike very much, 2 = dislike moderately, 3 = dislike slightly, 4 = neither like nor dislike, 5 = like slightly, 6 = like moderately, and 7 = like very much. The hedonic data obtained were categorised into 3 different groups, i.e., the T3B Overall Liking (Top 3 Box Rating), B3B Overall Liking (Bottom 3 Box Rating), and Neutral Overall Liking (Neutral) to determine the preferences of panellists. For JAR, the attributes and rating description are shown in Table 2. The JAR data obtained were analysed using penalty analysis (PA) consisting of 3 steps: (1) collapsing the 5-point JAR scale into 3 groups (too low, JAR, and too high), (2) calculating the percentage of consumers and the mean overall liking score for each group, and (3) calculating the mean drop by subtracting the mean overall liking score for the JAR group from the mean overall liking score of the “too high” or “too low” groups [41,42,50]. Penalty analysis was conducted to verify which of the attributes affected the acceptability of the product to a greater or lesser extent with the main objective being to improve the sensory quality of the product [53,54].

### 2.3. Data Analysis

The data obtained were analysed using tools XLSTAT (Version 2021.4; Addinsoft, Paris, France) and Microsoft Excel software. For the triangle test, ANOVA analysis and binomial table for paired preference two-tailed test were used to identify whether there was any significant difference on the overall liking and preference between the different products. Results from the overall acceptance and preference test were analysed using analysis of variance (ANOVA) and Tukey’s test to determine the significant differences between the products. Statistical differences with *p*-value lower than 0.05 were considered significant.

## 3. Results

### 3.1. Triangle Test

Table 3 lists the information for the triangle test where a significant difference (*p* < 0.05) between HPP and ESL milk at 95% confidence level yields a *p*-value < 0.0001. With guessing probability associated with three samples in a triangle test at only 1/3, 89.5% (34 out of 38) of panellists correctly identified the odd sample at *p* < 0.05. According to Lawless and Heymann [55], this test only required a minimum of 19 correct responses to imply a significant difference between the samples. Thus, this result shows that the detection of difference between HPP and ESL milk samples by panellists was not by chance. The proportion of discriminators, p_d_ in the population of interest, was calculated to express the sensory distance of the objects, with formula p_d_ = (p_c_ − p_g_)/(1 − p_g_), where p_c_ is the probability of a correct answer and p_g_ is the guessing probability, assuming panellists may comprise ignorant panellists who make guesses and discriminators who discriminate correctly and provide appropriate answers [56]. The proportion of discriminators p_d_, which was equal to 0.842 in this test, indicates that panellists had high discriminative ability. Further analysis involved the calculation of a lower limit and upper limit using an “exact” binomial interval, the Clopper–Pearson method, at confidence level 0.95. The midpoint of this interval is defined by the statistic test result of 33 panellists. As a result, this triangle test suggested that milk treated with a different approach or temperature was recognisable from its different taste and flavour. An earlier study by Grabowski et al. [57] revealed noticeable differences in the taste of HTST pasteurised milk (72.7 °C for 15–30 s) compared with ESL milk (125–127 °C for 2–4 s) where 80% of panellists were capable of distinguishing them clearly, as ESL milk had a noticeable cooked and sulphur flavour when compared with milk treated at a lower temperature. The key factor causing a change in flavour during the thermal processing of milk is correlated with Maillard reaction, lipid degradation, thermal denaturation of serum (whey) protein, and other proteins in the milk fat globule membrane [58]. During thermal processing, sugar (lactose) and protein (amino group) in milk undergo a Maillard reaction leading to the formation of flavour components such as Strecker aldehydes, sulphur- and nitrogen-containing compounds, maltol, and diacetyl which affect the taste of milk [59]. Lipid degradation during thermal processing causes off-flavour due to the liberation of volatile fatty acids, such as butyric acid, and oxidation of free or glyceride bound unsaturated fatty acids with subsequent formation of volatile compounds [60]. Thermal denaturation of the whey proteins, primarily β-lactoglobulin, and the proteinaceous material associated with the fat globule membrane generates free sulphydryls and volatile sulphides resulting in cooked, cabbage, and sulphur flavours in milk [61].

**Figure 1:**
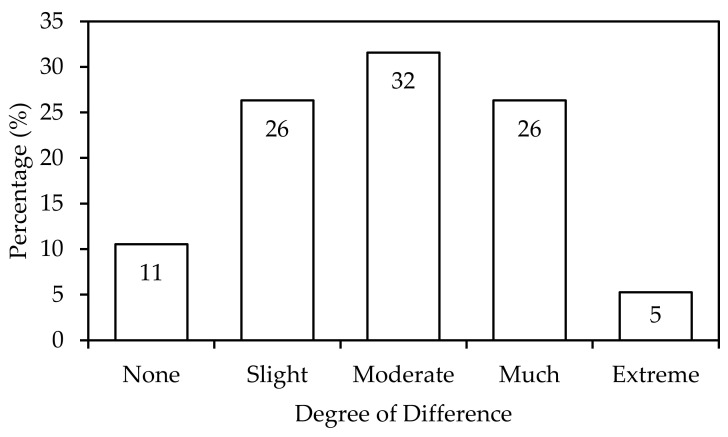
Selection of degree of difference “None”, “Slight”, “Moderate”,” Much”, and “Extreme” from the correct responses.

The 89.5% (34) panellists who successfully identified odd samples indicated the degree of difference between the milk samples as shown in Figure 1. Twelve panellists (32%) selected “Moderate”, followed by 10 panellists (26%) equally selecting “Slight” and “Much”, and only 2 panellists (5%) indicating “Extreme”. Table 4 shows the quantitative descriptive statistics analysis for the degree of difference at “Moderate” with a mean score of 1.895. The kurtosis value −0.689 indicates that this distribution is slightly less peaked than normal with a negative skewed distribution at the value of −0.049. Further analysis in terms of specific attributes of odour, appearance, mouthfeel, taste, and aftertaste are used to describe these differences in milk samples [49].

### 3.2. Acceptance and Preference Test

Table 5 shows the results of the overall liking evaluation on a 7-point hedonic scale. HPP milk scored higher on the T3B overall liking (Top 3 Box Rating) at 61% while ESL milk scored slightly lower at 53%. The average mean score of overall liking of HPP milk was also slightly higher at 4.66 when compared with ESL milk at 4.34. This result suggested a slight preference of HPP over ESL milk amongst the panellists. However, statistical analysis showed no significant difference (*p* > 0.05) between HPP and ESL milk in the overall liking mean score and preference test.

**Figure 2:**
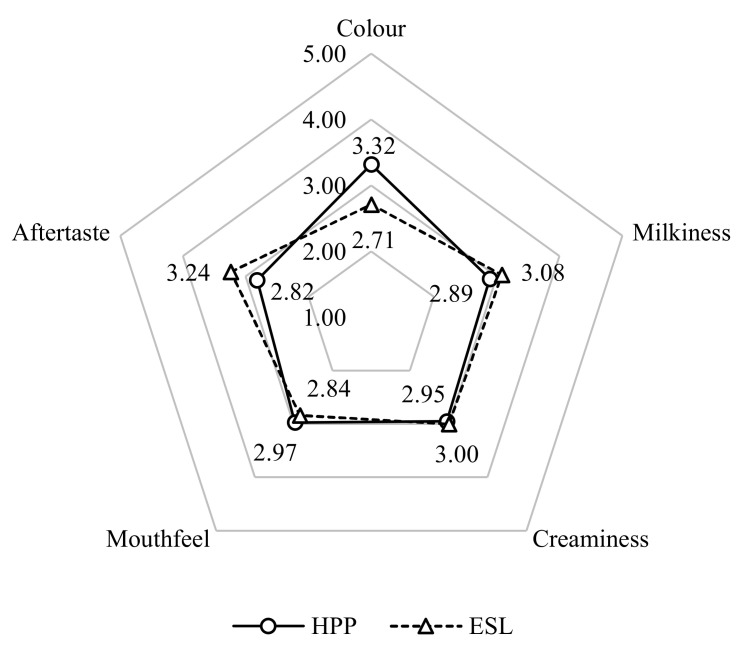
Comparison of average JAR scores between HPP and ESL milk.

The average JAR score rated by 38 panellists for the 5 sensory attributes of colour, milkiness, creaminess, mouthfeel, and aftertaste is presented in Figure 2. The aftertaste and milkiness of HPP fresh milk were slightly lower at a rating value of 2.82 ± 0.7 and 2.89 ± 0.9, respectively, as compared with ESL milk at a rating of 3.24 ± 1.1 and 3.08 ± 1.0, respectively. The mouthfeel of ESL milk was slightly thinner based on panellist acceptance with a rating of 2.84 ± 0.8. Panellists’ perception toward creaminess of HPP and ESL milk were almost similar with a score of 2.95 ± 0.9 and 3.00 ± 1.0, respectively. There were no significant differences (*p* > 0.05) in all attributes of milkiness (*p* = 0.419), creaminess (*p* = 0.814), mouthfeel (*p* = 0.504), and aftertaste (*p* = 0.060) except for colour (*p* = 0.0003). Similar results were reported by Liepa et al. [15] who found that there was a significant difference in colour (*p* = 0.022) of milk in the sensory properties acceptance evaluation of high pressure processed milk treated at 400 MPa and pasteurised milk treated at 78 °C for 15–20 s by 55 untrained panellists and 25 trained panellists. The high pressure processed milk was significantly lower in the L* value (colour parameter) and had an increased b* value (yellowness) causing the milk to look darker. This could be a result of pressure-induced micellar fragmentation which reduces the micellar size, causing milk to lose its ability to scatter light, becoming translucent and dark [47]. The sensory test conducted by Liu et al. [49] also reported noticeable differences between the flavour profile of HPP milk (600 MPa for 5 min) and LTLT pasteurised milk (63 °C for 30 min) in which the high pressure processed milk was the lowest in boiled and sweet odour, boiled taste, intensity, and cream taste while the LTLT-treated milk presented higher scores in boiled and sweet odour, intensity, cream taste, and off-taste. In terms of appearance, the same author reported a lower score for HPP over pasteurised milk in white appearance.

**Figure 3:**
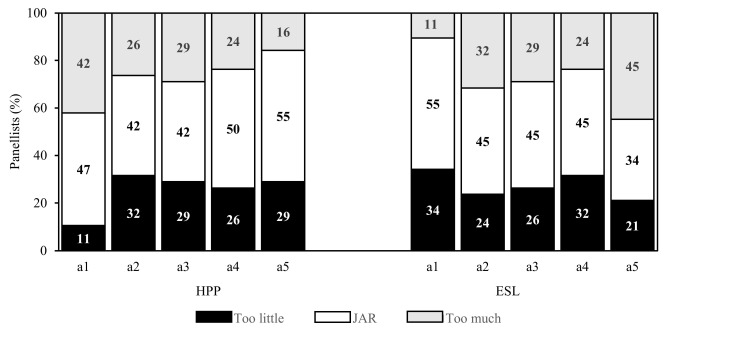
Percentage of panellists based on the collapsed JAR levels of the sensory attributes colour (a1), milkiness (a2), creaminess (a3), mouthfeel (a4), and aftertaste (a5) for HPP and ESL milk.

Figure 3 shows the results of the JAR evaluation where more panellists rated HPP milk samples as being the just-about-right level for mouthfeel and aftertaste while more panellists rated ESL milk samples as being the just-about-right level for colour, milkiness, and creaminess.

**Figure 4:**
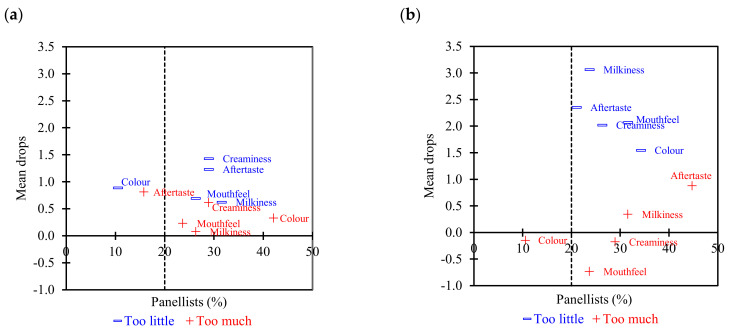
Mean drop plots for (**a**) HPP and (**b**) ESL milk. The dashed line represents the boundary of 20% of panellists.

Figure 4 shows the mean drop plot analysis that verifies the sensory attributes that have greater effects on product acceptability. The mean drop is the difference between the mean overall liking score for the just-about-right group and the mean overall liking score of the “too much” or “too little” categories. The vertical dashed line at 20% of *x*-axis (Selection) represents the boundary of respondents as a typical skew cut-off percentage used in the industry. If the number of responses is below the threshold of 20%, the penalisations are not taken into account as the percentage of responses are too small and this might not be reliable [41,51]. The sensory attributes which fall in the upper right quadrant (critical corner) of the chart are those that need most adjustment. Based on the mean drop plots in Figure 4, both creaminess and aftertaste fall at the critical corner indicating this product was perceived too little for creaminess and aftertaste. Hence, modification is required to increase creaminess and aftertaste of HPP milk. ESL milk on the other hand was perceived as low intensity for most sensory properties, except creaminess and mouthfeel. The attributes requiring improvement are depicted as “Penalty” in Table 6. The penalty is defined as the weighted difference between the means (mean of liking for JAR—mean of liking for the two other levels taken together) [62].

## 4. Discussion

In developing a new fresh milk product with better organoleptic properties and extended shelf life, HPP-treated milk was tested for consumer liking via sensory attributes in comparison with ESL milk. The results suggested that panellists liked the HPP milk more than the ESL milk from the higher percentage of panellists who selected the top three box (scores 4–7) for HPP milk instead of ESL milk. HPP milk scored better in overall organoleptic properties from the lower penalty values for most attributes including colour, milkiness, mouthfeel, and aftertaste with the exception of creaminess when compared with ESL milk. The noticeable change in colour in HPP milk was detected by panellists. This was verified by objective colour measurements instrumentally where the lightness, L-value, of HPP milk was lower at 90.8 as compared with ESL milk at 92.7 suggesting darker shades in HPP milk. The darker colour that appeared in HPP milk was in agreement with a previous study by Kim et al. [63] who found that the L-value of high pressure treated milk at 200 MPa for 10–30 min was significantly lower than that of raw milk. High pressure treatment induced modification of colloidal and emulsion components which led to colour changes of HPP-treated milk due to a different light scattering effect [20,64,65]. Pressure treatments are also reported to cause changes in colour and turbidity of milk due to the modified physicochemical properties of the casein micelles [66]. The disruptive effects of HPP on protein molecular forces such as hydrogen bonding, electrostatic, and hydrophobic lead to disaggregation of casein submicelles and dissociation of casein fractions from micellar casein to the soluble phase [65]. The compacted water molecules penetrate and hydrate milk micelle, causing dissociation of ion pairs that leads to the release of soluble calcium and phosphorus from micellar casein [66]. The disrupted micellar casein becomes smaller in size after releasing calcium and additional casein into milk serum [67]. The decrease in size of micellar casein further reduces the light scattering effect and results in a darker or more yellowish colour appearance in milk [15]. Although HPP milk was darker, the overall liking and preferences were not affected as the penalty was still lower by 0.712 at 0.439 when compared with ESL milk. The brighter appearance of ESL milk seems unfavourable to the panellists with a more significant penalty of 1.151 due to a wider range of scores in ESL than HPP milk despite ESL milk scoring well for Selection and Overall Liking (Table 6). This suggests that improvement is needed to reduce the brightness of ESL milk. ESL milk treated at a high temperature has a brighter colour due to serum protein denaturation and aggregation which increases the light scattering effect [15].

The taste evaluation of dairy products can be determined from tactile sensations produced in the mouth during tasting. These attributes include milkiness, creaminess, and mouthfeel. A milky sample can be described as a product with a soft, slippery, and smooth mouthfeel; creaminess as a product which provides a greasy, miscible, and thick mouthfeel [68]; while mouthfeel is a measure of the degree of thickness such as whether the sample provides a thin or thick sensation. The HPP milk scored lower in milkiness and creaminess but higher in mouthfeel properties when compared with ESL milk although both were found to be not significantly different. The perceptions of milkiness, creaminess, and mouthfeel of differently treated milk samples presumably were associated with changes in particle size of fat globules from different process methods and not due to the fat contents. Stratakos et al. [20] mentioned that the particle size of fat droplets present in dairy products is important in defining properties such as flavour release, mouthfeel, and emulsion stability. A previous study by Phillips et al. [69] showed that sensory scores for thickness, mouth coating, and residual mouth coating increase when fat content in milk increases. HPP up to 900 MPa did not cause significant changes to the lipid classes or fatty acid composition of milk fat as the neutral and polar lipids remained stable in the pressure range 250–900 MPa [70]. This was similar to thermal processing in which milk treated at 72 °C for at least 15 s did not cause significant changes to milk fat content [17]. Thermal processing has a minimum effect on milk fat whereby only the membrane of the fat globules with their heat-sensitive protein compounds undergo some modifications, affecting the agglomeration of fat globules and their creaming properties [71].

Aftertaste is the residual mouthfeel remaining in the mouth after swallowing the sample [72]. Aftertaste can be retained in the mouth from seconds up to a few minutes. This study shows that HPP milk had a lower sensory score for aftertaste as compared with the ESL milk. Approximately 29% of panellists had the perception that the aftertaste of HPP was too weak. Weak aftertaste concurrently reduced the overall liking and preferences of the panellists, resulting in a higher penalty. Porubcan and Vickers [73] reported that alternation of fat content, protein content, levels of defects such as oxidation, and other components of milk can change the sensory properties of milk, but no researchers have clarified how the aftertaste of milk is related to those changes. The same author established that aftertaste was associated with the volatile compounds produced during the light-activated oxidation of milk and these presumably were associated with the increased intensities of key attributes of “sour” and “dairy sour immediate”. This is consistent with the current findings where the majority of panellists voted ESL milk as giving a stronger aftertaste, probably due to a large amount of volatile components generated during high temperature treatment. These volatile components develop from a cooked, sulphur, or eggy taste in milk. Lee et al. [13] reported ESL milk had significantly higher sulphur or eggy flavours compared to HTST pasteurised milk which was treated at a lower temperature. The ESL milk had a higher penalty to overall liking for being thicker and having intense flavours and for being too thick. This suggests that the intensity of cooked flavour in ESL milk was likely an influential detractor from consumer acceptance.

## 5. Conclusions

There were noticeable differences between HPP and ESL milk as up to 89.5% of panellists selected the correct sample in the triangle test. The hedonic evaluation of overall liking of HPP and ESL milk showed that more panellists gave higher ratings on HPP milk. The JAR evaluation of acceptance and preference of the treated milk samples’ sensory properties showed no significant differences (*p* > 0.05) in all attributes which included milkiness, creaminess, mouthfeel, and aftertaste, with the exception of colour. The overall sensory properties of HPP-treated milk were evaluated higher for mouthfeel and aftertaste properties than ESL milk which scored higher for colour, milkiness, and creaminess. The colour differences were visible in HPP milk (darker colour) in comparison with ESL milk (lighter colour) as proven by literature. The penalty analysis suggested that HPP milk needs improvement for creaminess and aftertaste despite the later attribute still scoring better than ESL milk.

## Figures and Tables

**Table 1:** Main composition of raw, HPP, and ESL milk for sensory evaluation.

Composition (Per 100 g or mL)	Milk		
	Raw	HPP	ESL
Fat (g)	3.57 ± 0.12 ^a^	3.70 ± 0.15 ^a^	3.60 ± 0.06 ^a^
Protein (g)	3.10 ± 0.00 ^a^	3.27 ± 0.03 ^a^	3.27 ± 0.09 ^a^
Total Solids (mL)	12.07 ± 0.12 ^a^	12.27 ± 0.22 ^a^	12.13 ± 0.09 ^a^
Non-Fat Milk Solids (g)	8.40 ± 0.15 ^a^	8.50 ± 0.15 ^a^	8.33 ± 0.09 ^a^
Calcium (mL)	119.7 ± 3.2 ^a^	118.9 ± 0.7 ^a^	114.7 ± 2.3 ^a^
Phosphorus (mL)	73.6 ± 0.6 ^a^	73.2 ± 1.0 ^a^	71.0 ± 0.3 ^a^

^a^ Mean ± standard deviation marked with the same letters in the rows are not significantly different (*p* > 0.05).

**Table 2:** Attributes and 5-point just-about-right (JAR) scale used.

Sensory Attributes	Rating Description
Colour	1. Too Light 2. Slightly Light 3. Just Right 4. Slightly Dark 5. Too Dark
Milkiness	1. Not Milky Enough 2. Slightly Not Milky 3. Just Right 4. Slightly Milky 5. Too Milky
Creaminess	1. Not Creamy Enough 2. Slightly Not Creamy 3. Just Right 4. Slightly Creamy 5. Too Creamy
Mouthfeel	1. Too Thin 2. Slightly Thin 3. Just Right 4. Slightly Thick 5. Too Thick
Aftertaste	1. Too Weak 2. Slightly Weak 3. Just Right 4. Slightly Strong 5. Too Strong

**Table 3:** Summary of triangle test result.

Test	Triangle Test
Number of panellists	38
Probability of correct answers, p_c_	0.895
Guessing probability, p_g_	0.333
Proportion of discrimination, p_d_	0.842
Statistic	33.0
*p*-value	<0.0001
alpha	0.05

**Table 4:** Descriptive statistics analysis for “Moderate” difference between milk samples.

Mean	1.895
Standard Error	0.176
Median	2
Mode	2
Standard Deviation	1.085
Sample Variance	1.178
Kurtosis	−0.689
Skewness	−0.049
Range	4
Sum	72
Count	38
Confidence Level (95.0%)	0.357

**Table 5:** Summary for overall liking and preference test of HPP and ESL milk.

Overall Liking and Preference (7-Point Hedonic Scale)	Milk Samples	
	HPP	ESL
B3B Overall Liking (Bottom 3 Box Rating)	21%	26%
Neutral Overall Liking (Neutral)	18%	21%
T3B Overall Liking (Top 3 Box Rating)	61%	53%
Preference	47%	53%
Average Mean Score	4.66 ^a^	4.34 ^a^

^a^ Values marked with the same letters in the rows are not significantly different (*p* > 0.05).

**Table 6:** Penalty analysis table of HPP and ESL milk based on overall liking scores using the 7-point hedonic scale.

Milk Type	Variable	Level	Selection ^a^ (%)	Sum (Overall Liking) ^b^	Mean (Overall Liking) ^c^	Mean Drops ^d^	Penalty ^e^
HPP Milk		Too light	10.53	16.0	4.000	0.889	
	Colour	JAR	47.37	88.0	4.889		0.439
		Too dark	42.11	73.0	4.563	0.326	
		Not milky	31.58	51.0	4.250	0.625	
	Milkiness	JAR	42.11	78.0	4.875		0.375
		Too milky	26.32	48.0	4.800	0.075	
		Not creamy	28.95	42.0	3.818	1.432	
	Creaminess	JAR	42.11	84.0	5.250		1.023
		Too creamy	28.95	51.0	4.636	0.614	
		Too thin	26.32	42.0	4.200	0.695	
	Mouthfeel	JAR	50.00	93.0	4.895		0.474
		Too thick	23.68	42.0	4.667	0.228	
		Too weak	28.95	43.0	3.909	1.234	
	Aftertaste	JAR	55.26	108.0	5.143		1.084
		Too strong	15.79	26.0	4.333	0.810	
ESL Milk		Too light	34.21	43.0	3.308	1.549	
	Colour	JAR	55.26	102.0	4.857		1.151
		Too dark	10.53	20.0	5.000	−0.143	
		Not milky	23.68	19.0	2.111	3.065	
	Milkiness	JAR	44.74	88.0	5.176		1.510
		Too milky	31.58	58.0	4.833	0.343	
		Not creamy	26.32	28.0	2.800	2.024	
	Creaminess	JAR	44.74	82.0	4.824		0.871
		Too creamy	28.95	55.0	5.000	−0.176	
		Too thin	31.58	33.0	2.750	2.074	
	Mouthfeel	JAR	44.74	82.0	4.824		0.871
		Too thick	23.68	50.0	5.556	−0.732	
		Too weak	21.05	23.0	2.875	2.356	
	Aftertaste	JAR	34.21	68.0	5.231		1.351
		Too strong	44.74	74.0	4.353	0.878	

^a^ Selection % is the percentage of panellists who rated the milk at levels of too low, JAR, or too high. ^b^ Sum (Overall Liking) is the total score of panellists who rated the milk at levels of too low, JAR, or too high. ^c^ Mean (Overall Liking) is the average score for each level of too low, JAR, or too high. ^d^ Mean drops is the decrease in liking compared to the mean liking of those who rated the attribute as JAR. ^e^ Penalty is a weighted difference between means (mean liking of JAR category minus the mean liking for the other two levels (too low and too high taken together). Refer to the Appendix A for calculations.

## Data Availability

Data is contained within the article.

## Appendix A

Formula of mean drops:

The percentage of consumers in each of the 3 categories is calculated and corresponding mean liking (L_TL_, L_JAR_, L_TM_) scores for the “Too Little” (TL), “JAR”, and “Too Much” (TM) categories estimated. Mean drops is calculated as follows:

Mean drops TL = L_JAR_ − L_TL_

Mean drops TM = L_JAR_ − L_TM_

Formula of penalty:

The penalty is a weighted difference between the means (mean of liking for JAR—mean of liking for the two other levels taken together).

Example:

Penalty value for colour attribute of HPP milk = $\frac{\left(16\right)\left(0.889\right)+\left(73\right)\left(0.326\right)}{16+73}=0.439$.
